# Regulation of *FATTY ACID ELONGATION1* expression in embryonic and vascular tissues of *Brassica napus*

**DOI:** 10.1007/s11103-015-0309-y

**Published:** 2015-03-21

**Authors:** Hélène Chiron, Jeroen Wilmer, Marie-Odile Lucas, Nathalie Nesi, Michel Delseny, Martine Devic, Thomas J. Roscoe

**Affiliations:** 1 Laboratoire Genome et Developpement des Plantes, CNRS-UP UMR5096, Université de Perpignan, 52 Avenue Paul Alduy, 66860 Perpignan, France; 2 BIOGEMMA, Chappes Research Centre, Route d’Ennezat, 63720 Chappes, France; 3 UMR1349 INRA-Agrocampus Ouest-Université de Rennes, Institut de Génétique, Environnement et Protection des Plantes, BP 35327, 35653 Le Rheu Cedex, France; 4 Present Address: CNRS ERL5300 Epigenetic Regulation and Seed Development Group, IRD UMR232 DIADE, Institute de Recherche pour le Développment, 911 Avenue Agropolis, 34032 Montpellier Cedex 1, France

**Keywords:** *FATTY ACID ELONGATION1*, 3-*keto*-acylCoA Synthase, Transcriptional regulation, Very long chain fatty acids, Triacylglycerol, Barrier lipid, *Brassica napus*

## Abstract

The expression of the *FATTY ACID ELONGATION1* genes was characterised to provide insight into the regulation of very long chain fatty acid (VLCFA) biosynthesis in *Brassica napus* embryos. Each of the two rapeseed homoeologous genes (Bn-*FAE1.1* and Bn-*FAE1.2*) encoding isozymes of 3-*keto*-acylCoA synthase, a subunit of the cytoplasmic acyl-CoA elongase complex that controls the production of elongated fatty acids, are expressed predominantly in developing seeds. The proximal regions of the Bn-*FAE1.1* and Bn-*FAE1.2* promoters possess strong sequence identity suggesting that transcriptional control of expression is mediated by this region which contains putative *cis*-elements characteristic of those found in the promoters of genes expressed in embryo and endosperm. Histochemical staining of rapeseed lines expressing Bn-*FAE1.1* promoter:reporter gene fusions revealed a strong expression in the embryo cotyledon and axis throughout the maturation phase. Quantitative analyses revealed the region, −331 to −149, exerts a major control on cotyledon specific expression and the level of expression. A second region, −640 to −475, acts positively to enhance expression levels and extends expression of Bn-*FAE1.1* into the axis and hypocotyl but also acts negatively to repress expression in the root meristem. The expression of the Bn-*FAE1.1* gene was not restricted to the seed but was also detected in the vascular tissues of germinating seedlings and mature plants in the fascicular cambium tissue present in roots, stem and leaf petiole. We propose that Bn-*FAE1.1* expression in vascular tissue may contribute VLCFA for barrier lipid synthesis and reflects the ancestral function of *FAE1* encoded 3-*keto*-acylCoA synthase.

**Electronic supplementary material:**

The online version of this article (doi:10.1007/s11103-015-0309-y) contains supplementary material, which is available to authorized users.

## Introduction

Very long chain fatty acids (VLCFAs) possessing carbon chain lengths greater than C18 are chemically diverse components of myriad lipids in plants including suberin and epicuticular waxes and are present in sphingolipids of the endomembrane system and plasma membrane. VLCFA have been implicated in sphingolipid-mediated signalling of cell death during the Hypersensitive Response after pathogen infection and have been shown to be important in plant developmental processes including auxin transport, cell proliferation, epidermal cell–cell adhesion and also root development (reviewed Bach and Faure 2010). The seeds of many plant species including all studied crucifers and members of the *Limnanthes* genus contain VLCFAs as major components of their storage oils which are of interest as industrial feedstocks (Dyer et al. 2008).

VLCFAs are synthesised by an acyl-CoA elongase complex located at the endoplasmic reticulum using malonyl- and acyl-CoA substrates. Condensation of a 2-carbon unit derived from a malonyl-CoA donor to an acyl-CoA acceptor is catalysed by a 3-*keto*-acyl-CoA synthase (KCS) and the *keto* group is then removed from the extended acyl chain by a series of sequential reactions: 3-*keto* reduction to the 3-hydroxyacyl-CoA catalysed by a *keto*-acylCoA reductase (KCR), 3-hydroxydehydration via 3-hydoxyacyl-CoA dehydratase to the trans-2,3-enoyl-CoA and an enoyl reduction of the double bond via *trans* 2,3-enoyl-CoA reductase. In *Arabidopsis thaliana*, condensing enzymes are encoded by a large *FAE1*-like multigene family. Loss-of-function mutations in individual KCS encoding genes leads to depletion of VLCFAs of various chain lengths in either seeds (Kunst et al. 1992), cuticular waxes (Millar et al. 1999) or in root suberin (Lee et al. 2009). In *A. thaliana*, only one gene encoding a *keto*-acylCoA reductase (*KCR1*) and one gene encoding a 3-hydoxyacyl-CoA dehydratase (*PAS2*) have been shown to be essential (Beaudoin et al. 2009; Bach et al. 2008). To date, only one gene has been identified (*CER10*), encoding a *trans* 2,3-enoyl-CoA reductase, in which a loss-of-function mutation results in a lower VLCFA content but not lethality (Zheng et al. 2005).

Ectopic expression has revealed the paramount importance of condensing enzymes in conferring the capacity to synthesize VLCFA on diverse tissues and organs (Millar and Kunst 1997). In plants, it is probable that many elongase complexes exist in diverse organs, tissues and cell types that comprise common reductase and dehydratase subunits yet contain distinct condensing enzymes. Individual condensing enzymes possess distinct substrate preferences which permit the production of VLCFA of various chain lengths as precursors for distinct functional classes of lipids. *A. thaliana* possesses 21 genes that code for KCS enzymes distributed across the five chromosomes and certain are found in regions that have arisen by ancient and recent chromosomal segmental duplication and transposition events which have contributed to the expansion of the *KCS* family (reviewed Joubès et al. 2008). Based on analyses of substrate preferences and reaction products, capacity to complement yeast *elo* mutants and expression profiles, it is clear that redundancy and specialisation of function exists among members of the gene family. At this time, it is possible to assign function to ten KCS in Arabidopsis. Thus, KCS1, KCS6 and probably KCS5 are associated with the provision of VLCFA precursors for cuticular waxes. KCS2, KCS20 and KCS9 provide VLCFA precursors for wax and suberin production, the latter KCS9, is required for sphingolipids and phospholipid elongation to C24 chain length. KCS11 elongates C18–C20 for root membrane lipids. KCS10 and KCS13 seem to play more specific roles in development whereas KCS18/FAE1 provides VLCFA for assembly of seed storage lipids (reviewed Haslam and Kunst 2013; Jung et al. 2014).

In rapeseed, the accumulation of VLCFA correlated with a stimulation of the elongase activity during embryo development (Weselake and Taylor 1999) and similarly, in Arabidopsis seeds, VLCFA deposition closely paralleled *FAE1* transcriptional activity (Rossak et al. 2001) and coincided with the period of storage lipid accumulation (Baud et al. 2002). In Arabidopsis, the KCS responsible for the elongation of fatty acids destined for TAG synthesis in seeds is *FAE1* (James et al. 1995). The loss of elongase activity in seeds results in an altered triacylglycerol fatty acid composition as evidenced by the *fae1* mutants of *A. thaliana*, which have drastically reduced levels of VLCFAs (Kunst et al. 1992). This phenotype is similar to that exhibited by low erucic acid rapeseed, ‘canola’ cultivars. In the allotetraploid *Brassica napus*, successive elongation steps from oleoyl-CoA to erucoyl-CoA (C22:1) via eicosanoyl-CoA (C20:1) are each controlled by alleles at two loci, E1 and E2 corresponding to the structural genes encoding BnFAE1.1 and BnFAE1.*2*. The Bn-*FAE1.1* gene encoding a rapeseed ß-ketoacyl-CoA synthase was shown to be tightly linked to the corresponding E1 locus controlling seed erucic acid content accounting for 56.4 % of the variation in seed erucic acid content whereas the E2 locus (the respective Bn-*FAE1.2* gene) contributed 28.6 % of the variation in seed erucic acid content. The effects of the two loci combined explained 90.6 % of the trait variation with the residual variation possibly contributed by other genes subject to environmental effects or to the maternal genotype (Barret et al. 1998; Jourdren et al. 1996). The greater effect of the E1 locus on erucic acid content correlates with the higher expression level of Bn-FAE1.1 (Puyaubert et al. 2001). Studies in rapeseed have shown that the synthesis of erucic acid is controlled by elongase activities present in embryos of high erucic acid rapeseed but absent in embryos of LEAR varieties, a consequence of independent mutations in each of the *FAE1* genes that act post-transcriptionally. In certain LEAR varieties, Bn-FAE1.1 contains a phenylalanine residue substituted for a serine residue (F282S) and Bn-FAE1.2 is truncated immediately after a T472 K substitution which correlate with an absence of the KCS protein and dissociation of a high molecular weight complex that comprises the constituent condensing enzyme, reductases and dehydratase of the elongase holozyme (Roscoe et al. 2001).

Rapeseed varieties possessing high endogenous elongase activites produce greater quantities of VLCFA and their seed oils are enriched in VLCFA (Weier et al. 1997), strongly suggesting that elongase activity is one factor limiting VLCFA synthesis. It is clear that it is the tissue specific expression of individual members of the family of KCS isozymes that determines the capacity of a cell to accumulate VLCFA. An understanding of the regulation of expression of the *FAE1* gene will provide insight as to the factors that limit the accumulation of VLCFA in seeds and thus aid efforts to enhance the production of unusual fatty acids for use as industrial feedstocks in the seeds of cultivated plants. Since certain genes encoding enzymes of fatty acid modification (*FAE1*) and those of triacylglycerol synthesis (*TAG1*) show similar expression profiles during seed development (Baud and Lepiniec 2009), elucidating the mechanism of regulation of *FAE1* gene expression may be also expected to provide insight into the network controlling the accumulation of storage lipids in crucifer seeds. Our primary objectives in the present work were to isolate and describe the *Bn*-*FAE1* gene promoters and to define the regions conferring seed specificity and exerting control over the level of expression as a first step towards understanding the transcriptional regulation of the synthesis of VLCFA destined for the synthesis of storage lipids.

## Results

### Near identity of the proximal promoter regions of the rapeseed *FAE1* genes

The cDNAs CE8 and CE7 (Barret et al. 1998) correspond to the Bn-*FAE1.1* and Bn-*FAE1.2* genes respectively of *B. napus*. Alignment of the nucleotide sequences corresponding to the CE7 and CE8 open reading frames (ORF) revealed that CE7 and CE8 are 97.6 % identical and differ in 22 nucleotides. CE7 is homologous to *FAE1* of *Brassica rapa* (accession KF999615.1) differing in five nucleotides and is 99.6 % identical to *B. napus* accession AF274750 (Han et al. 2001). CE8 is homologous to *FAE1* of *Brassica oleracea* (accession AF490460.1) differing in 6 nucleotides. We conclude that CE7 and CE8 are encoded by the AA and by the CC respective parental genomes of *B. napus*. The recently published *B. napus* genome (Chalhoub et al. 2014; http://www.genoscope.cns.fr/brassicanapus/) confirms Bn-*FAE1.1* to be 99.7 % identical to a sequence present on chromosome A08 (start 10187495 end 10190875) and Bn-*FAE1.2* to be 99.8 % identical to a sequence present on chromosome C03 (start 55684055 end 55800803).

**Fig. 1:**
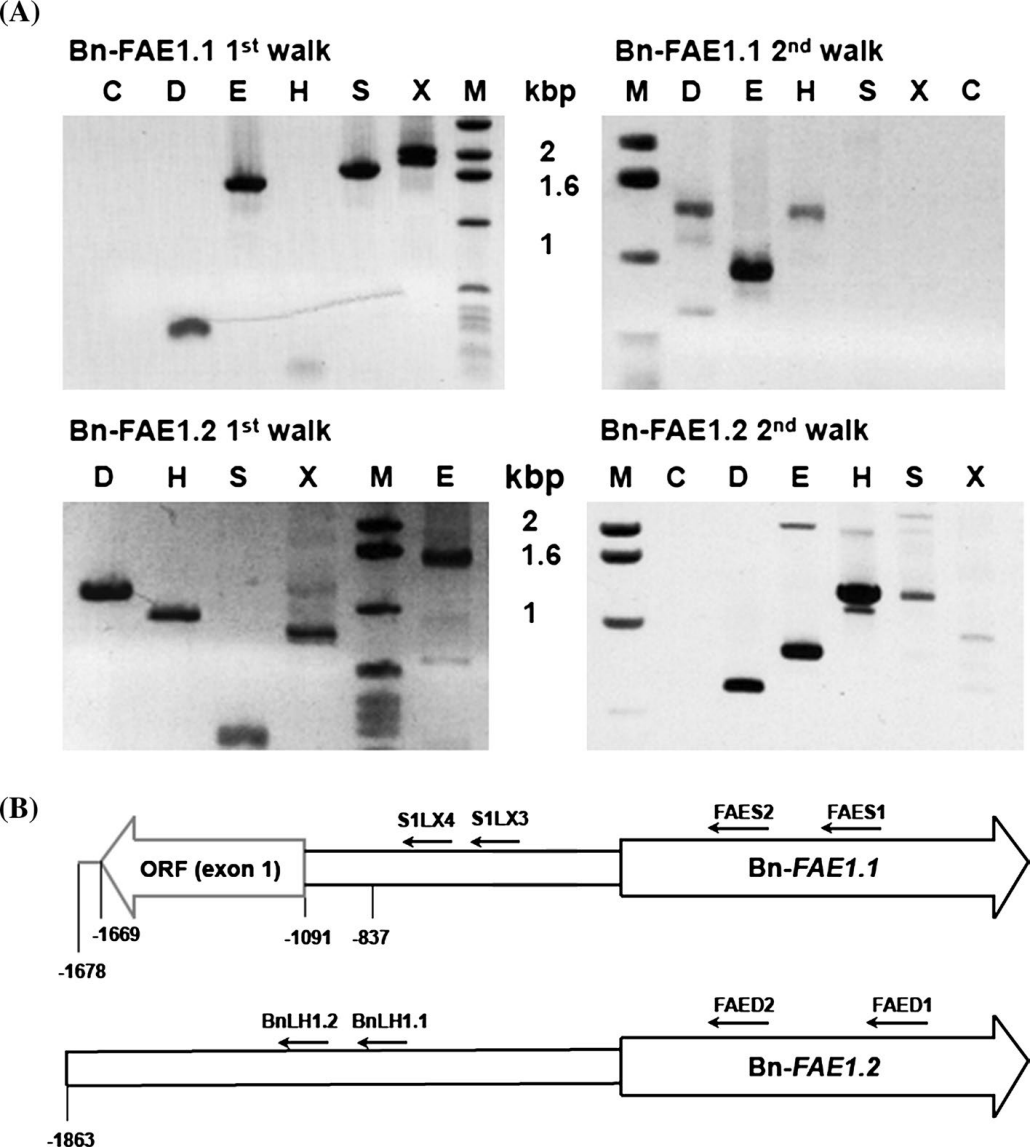
Isolation of the *B. napus FAE1* gene promoters. **a** PCR walking on genomic DNA from the Bn-*FAE1.1* and Bn-*FAE1.2* loci. Gene specific primers were used to amplify genomic fragments 5′ adjacent to the two Bn-*FAE1* coding sequences. Amplimers were obtained after nested PCR using FAES2 primer (Bn-*FAE1.1*, 1st walk) and S1X4 primer (Bn-*FAE1.1*, 2nd walk) and using FAED2 primer (Bn-*FAE1.2*, 1st walk) and LH1.2 primer (Bn-*FAE1.2*, 2nd walk). *Letters above lanes* indicate walk libraries used as templates, *C* control, *M* size marker. **b** Schematic representation of Bn-*FAE1* promoters showing positions of primers used for PCR walking. *Figures* indicate distances in nucleotides relative to the Adenine of the *FAE1* start codon

**Fig. 2:**
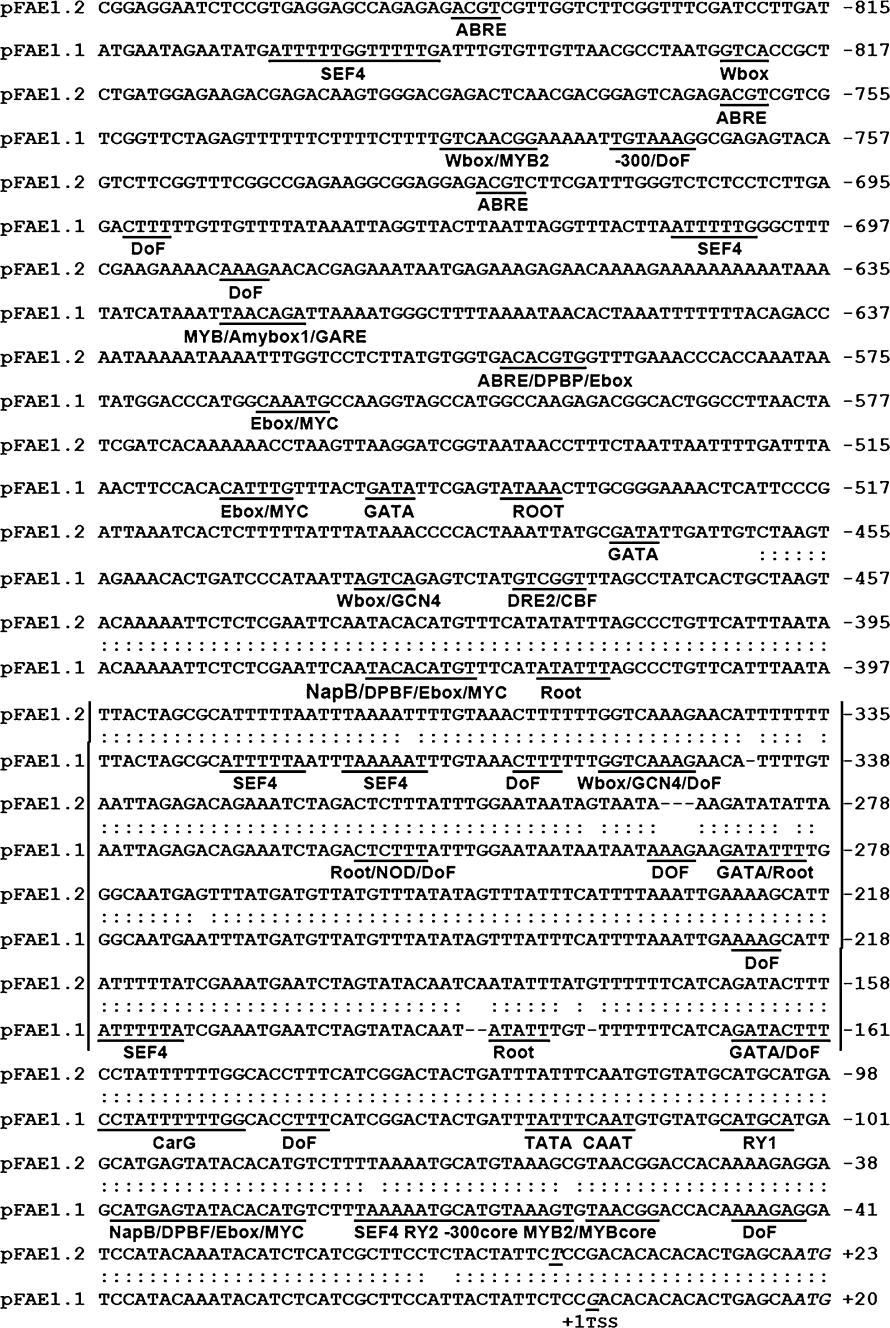
Alignments of the region 5′ upstream of the Bn-*FAE1* coding sequences. *pFAE1.1* and *pFAE1.2* correspond to the putative promoters of Bn-*FAE1.1* and Bn-*FAE1.2* respectively. Sequences are numbered with respect to the transcription start sites (TSS +1), *bold italicised* *C* in Bn-*FAE1.1* and *T* in Bn-*FAE1.2*. Sequence identity is indicated by *double dots*. Putative *cis*-elements are *underlined* and motif abbreviations are defined in the text. *Italicised* ATG corresponds to initiation codon. *Vertical bars* indicate *A*–*T* rich region

Since the rapeseed genome was not available prior to our experimentation, we exploited the nucleotide sequence variation that exists between the CE7 and CE8 cDNAs to derive gene-specific primers allowing the amplification of their respective promoters via a PCR walking strategy (Devic et al. 1997). Two successive promoter walks initiating from the Bn-*FAE1.1* coding sequence resulted in the amplification of several fragments allowing the assembly of a 1678 bp contiguous sequence (Bn-*pFAE1.1*) upstream from the initiation codon (Fig. 1). This Bn-*pFAE1.1* sequence (AA allele) shares 96 % identity over 486 bp at its 3′ extremity with the accession AF275254. Approximately 480 bp of the 5′ extremity of *Bn*-*pFAE1.1* corresponds to a protein coding sequence with strong homology to proteins present in cereals, reading divergently from the Bn-*FAE1.1* gene. The translated sequence shares 53 % identity and 66 % similarity with a predicted protein containing a Zinc Finger domain found in transposase/transcription factors and a hAT family dimerisation domain (accession number CDY09507) of *B. napus*. The 5′ extremity of the *Bn*-*pFAE1.1* sequence shares 75 % identity with an EST of *B. napus*, accession number, GR454096 confirming that this gene is expressed. PCR walks initiating from the Bn-*FAE1.2* gene encoding the CE8 cDNA allowed the isolation of an 1863 bp sequence (Bn-*pFAE1.2*) upstream from the ATG codon (Fig. 1). The *Bn*-*pFAE1.2* sequence is 99 % identical over 1433 bp to the *Bn*-*FAE* promoter sequence (CC allele, accession AF275254) of the cultivar Askari described by Han et al. (2001). Over 1678 base pairs, the Bn-*pFAE1.1* sequence shares 60 % identity with the Bn-*pFAE1.2* sequence although alignment of the two sequences revealed 96 % identity over 478 base pairs immediately upstream from the initiation codon (Fig. 2). There was no significant sequence homology beyond this proximal region.

### Analysis of the Bn-*FAE1* promoters

Primer extension analysis was performed to determine the transcription start site (TSS) of the Bn-*FAE1* genes (Supplementary figure 1). The TSS were established as the guanine at −17 of the Bn-*FAE1.1* promoter and the thymine at −20 of the Bn-*FAE1.2* with respect to the Adenine (+1) of the initiation codon (Fig. 2). The −17 position of the TSS coincides with the nucleotide defining the 5′ end of the majority of the longest ESTs corresponding to Brassica *FAE1* sequences in the Genbank database and therefore the 5′ non-translated sequences do not contain introns, a characteristic in common with the coding regions of *FAE1* genes. We conclude that *Bn*-*FAE1* promoters possess short 5′ non-translated sequences.

The Bn-*pFAE1* sequences were analysed for the presence of putative *cis*-elements by searching PLACE (http://www.dna.affrc.go.jp/PLACE/) and PLANTCARE (http://bioinformatics.psb.ugent.be/webtools/plantcare/html/). There were no consensus sequences corresponding to a TATA box nor a CCAAT box present in the proximity of the TSS (Fig. 2). The *Bn*-*FAE1* putative promoters are characterised by the presence of sequences corresponding to *cis*-elements associated with embryo and endosperm specific expression. These include putative binding sites for DNA one finger binding (DOF) motifs (AAAG) occurring eight times in the proximal promoter. Two RY motifs (CATGCATG) are also present. The E-box motif (CANNTG), occurs up to four times. Also present are motifs corresponding to the SEF4 motif (ATTTTTA) occurring five times, two −300 elements (TGTAAAG) as well as three binding sites for MYB factors (YAACKG) and a GCN4-like element (GTCA core) each occurring in storage protein promoters in cereals. A CArG motif (CC-A/Tx8-GG) is present in both Bn-*FAE1* promoters and is the binding site for AGAMOUS-Like 15 (AGL15) which controls the expression of regulators of embryogenesis and also genes encoding proteins involved in gibberellic acid and auxin metabolism. These putative elements occur predominantly within the conserved proximal region common to each promoter on either strand and several also occur further upstream. The region upstream of these motifs between −349 and −168, common to both promoters, is 79 % AT rich.

In the distal region there are motifs corresponding to putative *cis*-elements implicated in responses to biotic and abiotic stress, hormonal control and regulation of tissue specific expression. That such elements may be important in the regulation of *FAE1* genes is suggested by searches of the Arabidopsis expression browser (http://bar.utoronto.ca/efp/cgi-bin/efpWeb.cgi). These include putative W-box motifs mediating defence signalling in response to pathogens or elicitors also implicated as regulators of abscisic acid and of giberellin signalling and a gibberelic acid responsive element (GARE1), a drought responsive element (ACCGAC) together with MYB2 consensus (C/TAACG/T) and MYC (CANNTG) motifs, that are also implicated in ABA signalling are also present. The Bn-*FAE1.2* distal promoter contains three ABRE-like elements (abscissic acid response element), whereas in the Bn-*FAE1.1* promoter a single ABRE, occurs at −1133. ABRE motifs are found in promoters of dehydration-induced genes and in seed expressed genes, Motifs controlling root specific expression occur in the proximal and distal promoters.

### The Bn-*FAE1* genes are expressed during embryo maturation

**Fig. 3:**
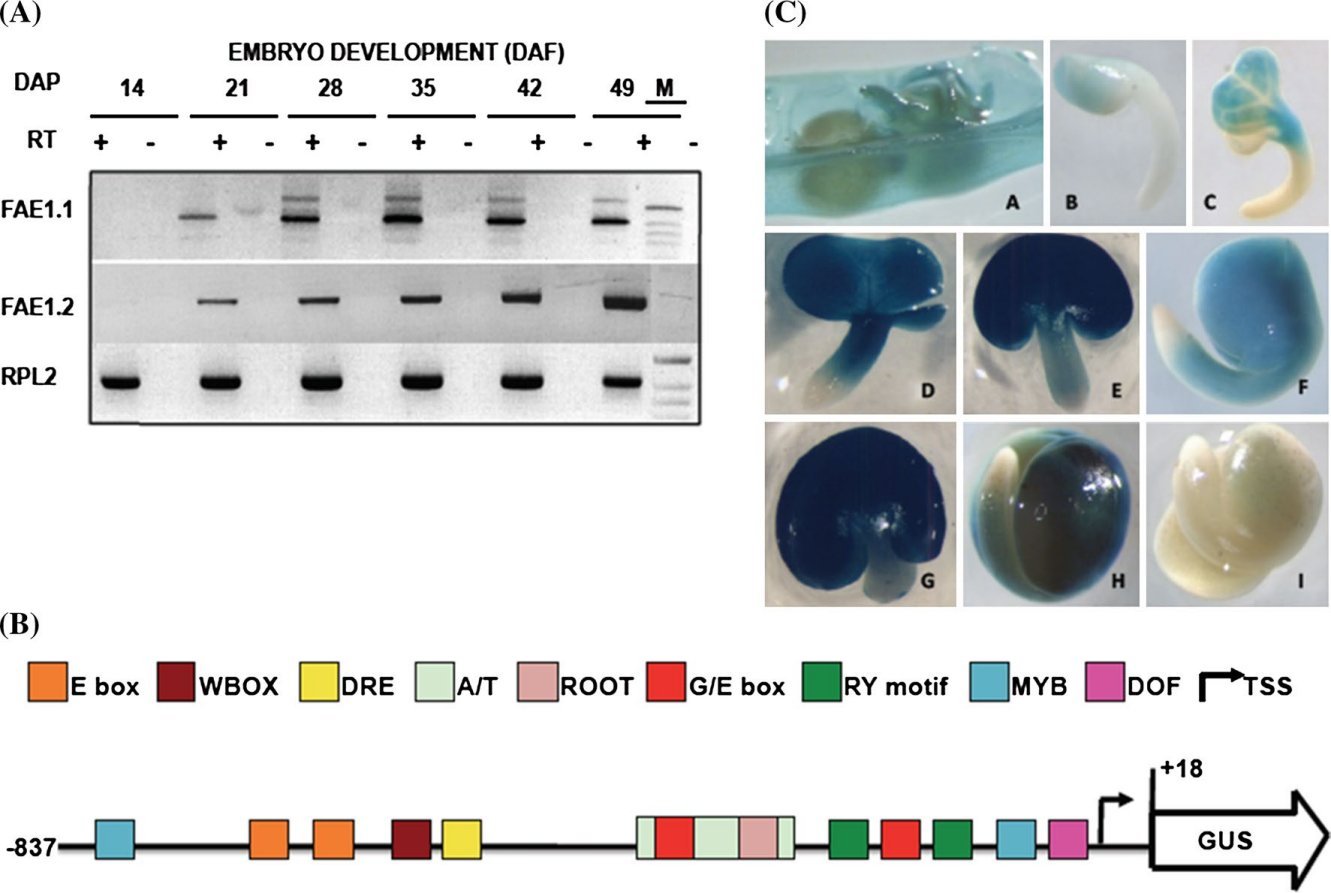
Bn-*FAE1.1* expression in rapeseed embryos. **a** RT-PCR analysis of Bn-*FAE1.1* and Bn-*FAE1.2* gene expression during embryo development. *Numbers* are stage of embryo development in days after fertilisation (DAF), expression of *RPL2* gene controls quantity of RNA. *RT* presence or absence of reverse transcriptase. *M* size marker co-migrated in 49 DAP negative control lane. **b** Structure of the Bn-*FAE1.1* promoter. A sequence of 857 bp amplified from the region upstream of the Bn-*FAE1.1* open reading frame was fused to the *UIDA* (GUS) gene. The locations of several putative *cis*-element landmarks are indicated. The transcription start site is indicated by *arrow*, adenine of start codon is positioned at +18. **c** Bn-*FAE1.1* expression in reproductive tissues of Bn-p*FAE1.1*_857_ *::GUS* lines. *A* opened young silique. *B*, *C* early cotyledonary stage embryos. *D*–*F* mid-cotyledonary stage embryos. *G*–*I* late cotyledonary stage embryos. Embryo in *I* is from a non-transformed plant

To verify the expression of the Bn-*FAE1* genes during seed development PCR primer pairs capable of amplifying either gene specific transcripts were designed. Semi-quantitative RT-PCR using RNA isolated from developing rapeseed siliques revealed that both Bn-*FAE1* genes were expressed between 21 and 49 days after pollination and that the highest level of expression for Bn-*FAE1.1* occurred between 28 and 42 days DAP whereas for Bn-*FAE1.2* this occurred later at 49 DAF (Fig. 3a). ESTs corresponding to Bn-*FAE1* genes have been identified in seed and embryo cDNA libraries of *B. napus*, *B. rapa* and *B. oleracea*. Since Bn-*FAE1.1* (AA allele) expression coincides more closely with VLCFA and TAG accumulation during seed development and exerts greater genetic control over VLCFA content in seeds we decided to characterise the promoter of this gene in detail. The Bn-*FAE1.1* gene is delimited by the 5′ flanking region of the upstream adjacent gene (Fig. 1b). In consequence, the regulatory region may be contained within the sequence −1091 upstream of the Bn-*FAE1.1* open reading frame and possibly within the 470 bp proximal promoter region common to each Bn-*FAE1* gene (Fig. 2). To define the regulatory regions controlling expression of this gene in seeds, a sequence of 857 bp upstream of the coding region was fused to a GUS reporter gene (Fig. 3b). The promoter:reporter construction was used to transform *B. napus* and seeds from T2 plants harbouring single copy constructions from 6 independent transformation events were examined for GUS staining (Fig. 3c). In seeds containing each of the D3 constructs (−837 to +20 promoter), staining was evident in the carpel, septum and funiculus of siliques containing torpedo stage embryos (Fig. 3c, panel A). The onset of GUS expression appeared in the cotyledons but not the vascular tissue of early cotyledonary stage embryos (Fig. 3c, panels B, C) and extended to the axis of the mid-cotyledonary stage embryo (Fig. 3c, panels D–F). Staining increased in these tissues and extended to the hypocotyl in late cotyledonary stage embryos. Staining in the hypocotyl weakened during embryo development to maturation (Fig. 3c, panels G, H). Maximum staining was evident between 28 and 35 DAF and was persistent to maturation but was not observed in the root tip (Fig. 3C, panel H). There was no consistent evidence of GUS staining in the endosperm at early development although staining was observed infrequently on the inside of the seed coat probably corresponding to the endosperm aleurone cell layer. Staining was absent from seeds of all non-transformed control plants (Fig. 3c, panel I). Since the promoter activity during embryo development corresponds to the transcript abundance detected by RT-PCR and the pattern of GUS staining corresponds closely with that observed by Rossak et al. (2001) for the 934 bp promoter of At-*FAE1* we conclude that the 857 bp promoter of Bn-*FAE1.1* contains all the elements necessary for expression in seeds.

### Upstream activating sequences exert control over the expression level of Bn-*FAE1.1*

**Fig. 4:**
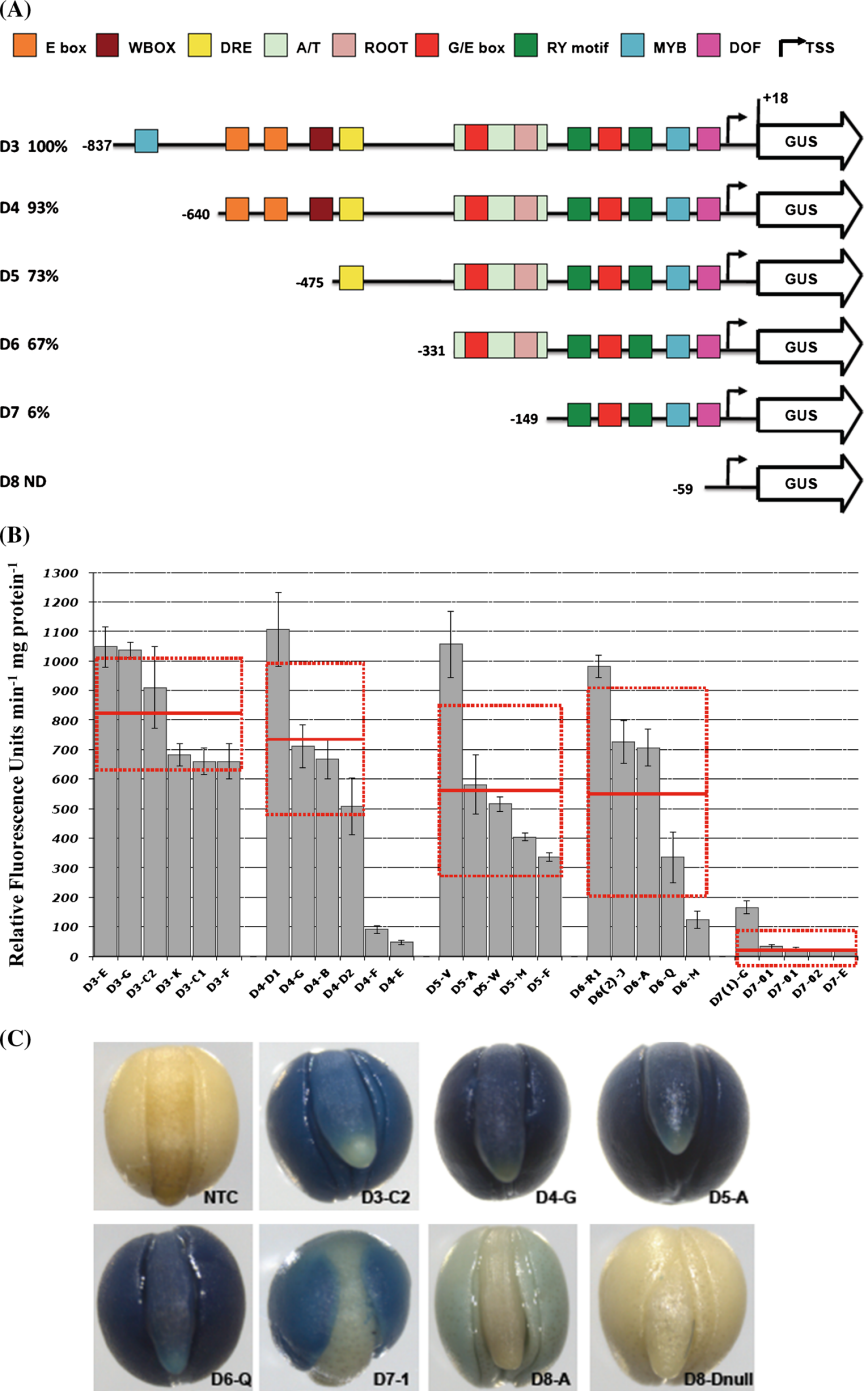
Expression of Bn-*FAE1.1* in embryos of *B. napus* promoter-deletion lines. **a** Bn-*FAE1.1* promoter-deletion constructs. Fragments amplified from the region upstream of the Bn-*FAE1.1* coding sequence were used to generate a series of promoter fragments fused to the *GUS* reporter gene named *D3*–*D8*. Positions of deletion end-points relative to the transcription start site are indicated by *numbers*. Percentage values are relative to activity of D3 deletion. The locations of putative *cis*-elements are indicated as *blocks*. *ND* activity not detected. **b** Reporter gene activity in Bn-*FAE1.1* promoter-deletion lines. GUS activity was quantified in protein extracts isolated from mature seeds. Extracts were prepared from three seeds of four independent plants (indicated by *histogram* and *error bars*) and from each of five or six transformation events corresponding to each promoter deletion. Enzyme and protein assays were performed in duplicate for each of the three biological replicates. *Red bars* represent overall means and standard errors for all plants of each event for each deletion. **c** Bn-*FAE1.1* expression in mature embryos. *D3*–*D8* corresponds to promoter deletion series indicated in **a**, plants from individual transformation events are identified by *letters* or *numbers* after the hyphen. NTC is a non-transformed control plant and null indicates a wild type seed segregating in T1 plants

To define the regulatory sequences that control the expression of the Bn-*FAE1.1* gene in seeds, a series of promoter deletions fused to GUS were constructed, based on the bioinformatics analysis reported in “Results” section (Fig. 4a). The Bn-*FAE1.1* promoter constructions included the short 5′ UTR (17 bp) were used to transform *B. napus*. Up to eight independent primary tranformants were regenerated for each construction. A total of 686 T2 plants harbouring these constructs were obtained and were analysed by Q-PCR to confirm the presence and transgene copy number. A set of 106 homozygous T2 plants containing a single T-DNA insertion, together with several non-transformed control plants were grown to produce tissue and seed samples for GUS histochemical staining. Seeds from the majority of 63 transformed plants examined exhibited GUS staining. Although the levels of reporter activity varied, no consistent differences were observed in the spatial or temporal pattern of expression among independent lines expressing the same construct.

Reporter gene activity was quantified in order to identify regions of the promoter important in controlling the level of Bn-*FAE1.1* expression in the embryo. GUS activity was measured using extracts obtained from T3 seeds containing late cotyledonary stage embryos from up to four individual plants for each of the independent transformation events representing each of the six promoter deletions (a total of 27 events) together with extracts from seeds of two non-transformed plants (Fig. 4b). Reporter activity was detected in all seeds from all lines that contained Bn-p*FAE1.1::*reporter constructs except for the D8 deletion. Progressive truncation of the Bn-*FAE1.1* promoter region resulted in a decrease in GUS reporter gene activity to minimum levels with the D7 deletion, −149 to +17. Between the −837 and +17 (D3) and the −149 to +17 (D7) deletions, GUS activity decreased approximately 40-fold. There was no significant difference in expression levels of Bn-*FAE1.1* between the D3 and the D4 deletions suggesting that all the elements necessary for the correct level of expression of Bn-*FAE1.1* are present within the region 640 bp proximal to the transcription start site. In contrast, the loss of an additional 164 bp fragment (D5 deletion) and a 181 bp fragment (D7 deletion) resulted in a decrease of 27 and 94 % respectively in GUS activity compared to D3 indicating the loss of elements that exert a positive control over the level of Bn-*FAE1* expression and may be considered as Upstream Activating Sequences (UAS). The 164 bp sequence was named as UAS2 and the 181 bp sequence as UAS1. The presence of low but significant GUS activity in seeds with the D7 promoter fragment suggests the presence of positive control elements in the promoter region between −149 and +17 able to promote transcription. In contrast, the activity of the −59 to +17 truncations (D8 deletion), was negligible under the conditions of the assay (data not shown) and was not significantly different from that of the non-transformed control plants which exhibited activities that were twofold–fourfold less than those obtained with the D7 construct.

### Specific regions of the Bn-*FAE1.1* promoter control expression in distinct domains of the embryo

The intensity of GUS staining observed in embryos obtained from each of the Bn-*FAE.1.1* promoter deletion transformants reflected the variation in GUS activity among the individual transformation events. In addition to the decrease in the level of reporter gene activity observed as the Bn-*FAE1.1* promoter was truncated, the pattern of GUS staining within the different regions of the embryo was modified (Fig. 4c). Essentially, all promoter fragments from −857 to −149 in length were able to confer GUS expression in the cotyledons. Staining, when present, was always stronger in the cotyledons than in the hypocotyl. The region between −857 and −475 conferred a strong staining in the cotyledon, hypocotyl and upper part of the root but not the root tip (Fig. 4c, panels D3-C2; D4-G). Between the −475 and −331 deletions, GUS staining was evident in all tissues of the embryo including the root tip (Fig. 4c, panel D5A; D6-Q) indicating the loss of a negative regulatory element present between −640 and −475 controlling expression in these specific root tissues. The region between −331 and −149 conferred a moderate intensity of staining in the cotyledons but none in the hypocotyl axis and the root (Fig. 4c, panel D7-1). In contrast, the D7 embryos showed a much weaker staining restricted to the cotyledons and predominant at centre of the cotyledons. The shortest deletion, −59 to +17, showed extremely weak staining even after prolonged incubation to 48 h that was restricted to the cotyledons only (Fig. 4c, panel D8-A). No GUS staining was evident in the embryo of seeds of non-transformed plants (Fig. 4c, panels NTC; D8-null).

### The Bn-*FAE1.1* gene is expressed in vascular tissues

**Fig. 5:**
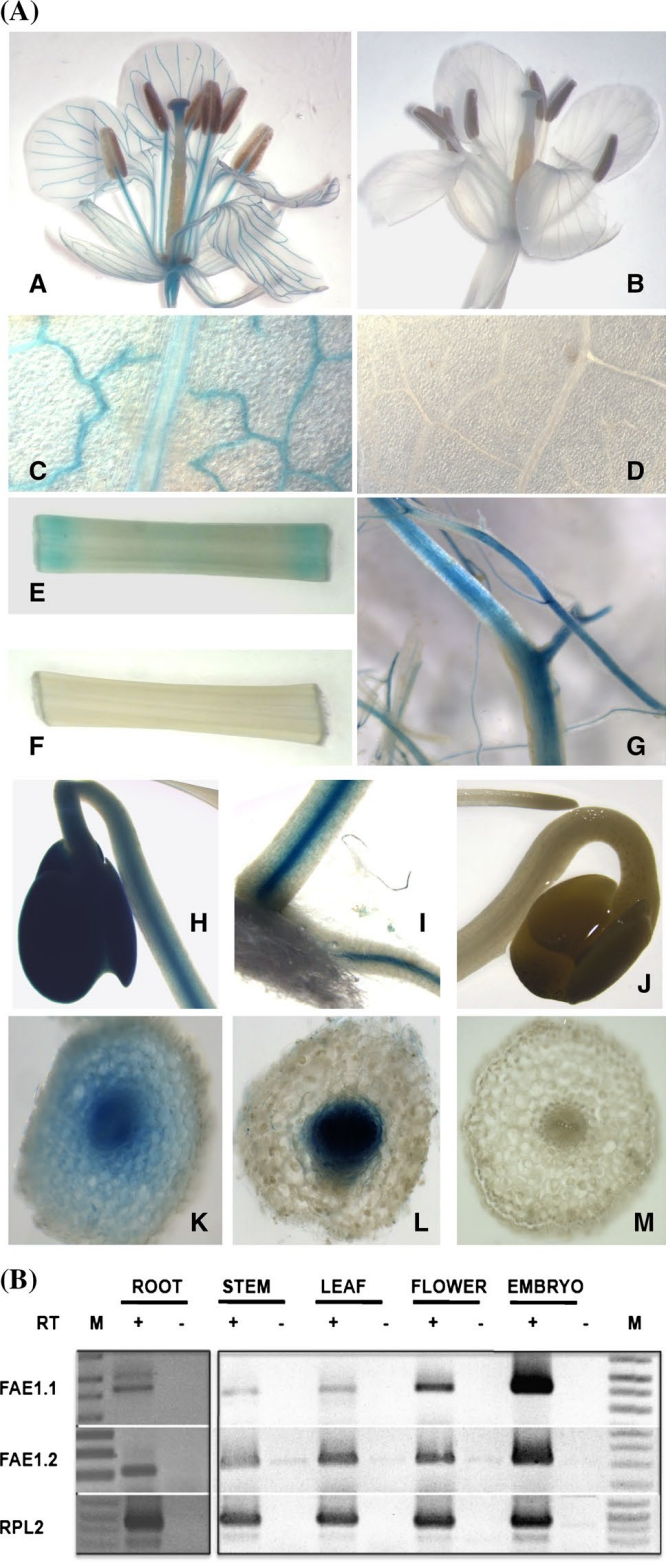
Bn-*FAE1.1* expression in vegetative tissues. **a** Reporter gene activity was visualised in tissues of flowering *Brassica napus* plants harbouring the Bn-p*FAE1.1*_837_ *::GUS* construct. *A*, *B* Open flower. *C*, *D* young expanding leaf. *E*, *F* section of primary stem. *G* primary and secondary roots. *H*–*J* germinating seedlings, *H* cotyledons and hypocotyl, *I* hypocotyl and primary root at the collet region of the seedling shown in *H*. *J* cotyledons, hypocotyl and root tip. *K* hand cut transverse section of hypocotyl of seedling shown in *H*. *L* hand cut transverse section of root of seedling shown in *I*. *M* Hand cut transverse section of root shown in *J*. *B*, *D*, *F*, *J*, *M* tissues from non-transformed control plants. **b** RT-PCR analysis of Bn-*FAE1.1* and Bn-*FAE1.2* gene expression in tissues of *B. napus*. Expression of *RPL2* controls quantity of RNA template. *RT* presence or absence of reverse transcriptase, *M* indicates size marker

Remarkably, unlike Arabidopsis, the expression of *FAE1.1* gene was not restricted to the embryo since the promoter was also active in the root and aerial vascular tissues of *B. napus*. In plants containing the D3 and D4 promoter fragments, GUS staining was evident in the vascular tissues of open flowers, particularly in sepals, petals and in anther filaments (Fig. 5a, panel A) and in young leaves, especially in differentiating vasculature but declining in mature vasculature (compare veins with midrib, Fig. 5a, panel C). GUS staining was evident in the vascular tissues of excised stem fragments, appearing more rapidly and stronger at the sites of wounding caused by sectioning the organ (Fig. 5a, panel E). The margins of leaf sections were also stained at the sites of excision (not shown). GUS staining was strong in mature primary and secondary roots but absent in young roots and root tips (Fig. 5a, panel G). Staining was also evident in the cotyledons, the hypocotyl and roots of germinating seedlings (Fig. 5a, panels H, I) and was localised to the central stele (Fig. 5a, panels K, L). Staining in vascular tissues was found in all Bn-*FAE1.1* promoter-deletion lines, including the D8 minimal promoter (−59 to +17), although the intensity of staining decreased strongly as the promoter was progressively truncated. With the exception of roots, visualisation of GUS activity in vascular tissues required prolonged staining (24 h) compared to embryos. No GUS activity was detected in any vascular tissue of non-transformed plants (Fig. 5a, panels 5B, D, F, J, M).

These observations contrast with the reports of Rossak et al. (2001) using the 934 bp At-*FAE1*promoter:GUS fusion and Han et al. (2001) using a 1.4kbp Bn-*FAE1.2* promoter:GUS fusion who state that *FAE1* promoter activity was absent from vegetative tissues of Arabidopsis and *B. napus* respectively. To independently confirm the profile of promoter activity we performed RT-PCR using RNA isolated from vegetative tissues. Expression of both Bn-*FAE1* genes was detected in root, stem, leaf and flower at lower levels compared to the level expressed in 28 DAF embryos (Fig. 5b). ESTs corresponding to Bn-*FAE1* genes are present in germinating rapeseed libraries and are present in a radish flower cDNA library (accession FY432873).

### Localisation of Bn-*FAE1.1* gene expression in vascular tissues

**Fig. 6:**
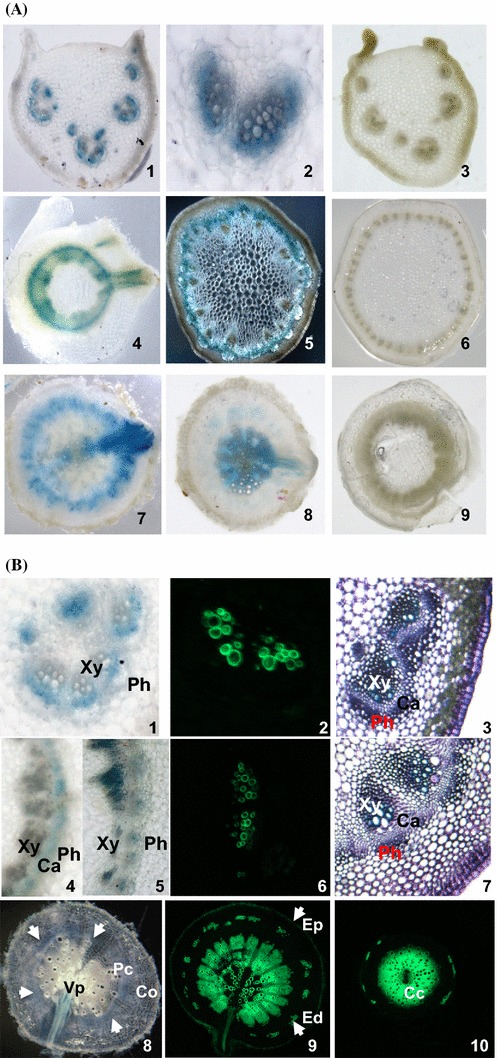
Localisation of Bn-*FAE1.1* expression in vascular tissues of *B. napus*. **a** GUS activity was visualised in hand-cut transverse sections of roots, stems and leaf petioles of young *B. napus* plants transformed with the Bn-p*FAE1.1*_837_:*GUS* construct. *1*–*3* Leaf petioles showing staining restricted to vascular bundles. *4*–*6* Stems. *4* Upper stem with emergent leaf vasculature. *5* Mid stem, showing fasicular staining in *dark field*. *7*–*9* Roots. *7* Mid root with emerging lateral root. *8* Lower root. *3*, *6*, *9* Stained sections from a non-transformed control plant. **b** Vibratome-cut transverse sections (100 µ) of leaf petioles (*1*–*3*) *1* GUS staining in zone between xylem and phloem; mid stem (*4*–*7*); mid root (*8*, *9*), lower root (*10*). Transverse sections *3* and *7* are stained with toluidine blue. Sections *2*, *6*, *9* and *10* are stained with berberine hemi-sulphate to visualise suberin aromatic component, counter-stained with aniline and visualised under UV light. Transverse sections *1*, *4*, *5* and *8* are stained for GUS activity. *White arrows* indicate extent of annulus of GUS staining in *8*. *Co* cortex, *Ed* endodermis, *Ep* epidermis, *Pc* pericycle, *Cc* central cycle, *Vp* vascular pole, *Xy* Xylem, *Ca* vascular cambium, *Ph* phloem

To describe the vascular expression of Bn-*FAE1.1* in greater detail, transverse sections of leaf petioles, stem and root were hand-cut and stained for GUS activity. Staining was present in vascular bundles of leaf petioles in cells surrounding the xylem (Fig. 6a, panels 1, 2). In young stem, near the plant apex staining was evident as a radial zone but was continuous with the vascular bundles from leaf petioles as evident from staining at the fusion of leaf to stem (Fig. 6a, panel 4). In mature stem, staining was localised as a radial band between the cortex and the endoderm (Fig. 6a, panel 5). Staining was evident as a radial band in the pericycle at mid distance along the primary root and was also present at the sites where the vascular tissue of secondary roots fused with that of the primary root (Fig. 6a, panel 7) and was strong in the central cycle (or stele) of young primary roots (Fig. 6a, panel 8). Staining was absent in sections cut from non-transformed plants (Fig. 6a, panels 3, 6, 9). We concluded that the Bn-*FAE1.1* gene is expressed in vascular tissue from differentiating roots through to the near apex of the stem. Detection of GUS staining in floral and young leaf vascular tissues indicates continuity of Bn-*FAE1.1* expression from root to all aerial vasculature.

Since the *keto*-acylCoA synthase encoded by *FAE1* controls the production of VLCFA we hypothesised that in vascular tissue these elongated fatty acids may constitute precursors for suberin or a suberin-like polymer to produce an impermeable barrier in aerial vascular tissue. To identify the precise cell types expressing the reporter gene, thin sections were cut using a vibratome and were stained for GUS activity or for suberin. In 100 µm sections GUS staining was weak in all tissues examined. Berberine hemisulphate staining was used to detect the aliphatic and phenolic components of suberin since attempts to the aliphatic component of suberin using fluorol yellow were unsuccessful. The co-localisation of GUS and suberin directly in the same sections was not possible due to fluorescence quenching. In petioles, GUS staining was intrafascicular and frequently restricted to the cells lying between the xylem and the phloem (Fig. 6b, panel 1) corresponding to the vascular cambium region in the section stained with toluidine blue (Fig. 6b, panel 3). In the stem, GUS staining was also frequently observed in the vascular cambium (Fig. 6b, panel 4) but was also observed in both the xylem and phloem (Fig. 6b, panel 5) as evident with toluidine blue staining (Fig. 6b, panel 7). In petioles (Fig. 6b, panel 2) and stems (Fig. 6b, panel 6) berberine staining was associated with the secondary xylem and was strongly in excess of cell wall auto-fluorescence. In mid roots GUS staining was present as a radial band in the pericycle between the cortex and the stele (Fig. 6a, panel 7; 6b, panel 8) and was approximately coincident with berberine staining (Fig. 6b, panel 9). In younger roots, GUS staining was present in the vascular cylinder (Fig. 6a, panel 8) and was directly coincident with berberine staining (Fig. 6b, panel 10). We conclude that Bn-*FAE1.1* is expressed in the meristem and differentiating zone of the vascular tissue, the cambium and secondary xylem and possibly secondary phloem and is co-incident with tissues undergoing suberin deposition.

## Discussion

In this study, analyses of the Bn-*FAE1* promoters revealed the presence of putative *cis*-elements including RY repeats, G and E boxes, DOF and MYB motifs which occur in the promoters of genes associated with the synthesis of seed reserves (Vicente-Carbajosa and Carbonero 2005). We show that the proximal region of the Bn-*FAE1.1* promoter contains all the *cis*-acting elements necessary for high level expression within the cotyledons, the principle site of oil accumulation in rapeseed embryos (Li et al. 2006) and we identify regions of the promoter that determine expression within the cotyledons, hypocotyl and root tip. By defining the regions of the promoter which control temporal and tissue specific expression of Bn-*FAE1* this research has provided insight as to the transcriptional control of VLCFA accumulation in the seed. In documenting the similarities and differences in tissue specific expression, overall promoter structure and *cis*-elements between Bn-*FAE1* with seed expressed genes that control reserve synthesis, this work has provided a basis for understanding the regulation of genes that share similar expression profiles including those controlling triacylglycerol assembly and storage. Remarkably, Bn-*FAE1.1* expression is not restricted to the seed but is also expressed in vascular tissue throughout the plant, suggesting functions for Bn-*FAE1* genes additional to the production of VLCFA as a component of triacylglycerol.

### An A/T rich UAS is a major determinant of the level of Bn-*FAE1* expression in embryos

The Bn-*FAE1* genes do not contain introns in the coding sequence nor in the 5′ non-translated sequence therefore the level of expression of the Bn-*FAE1* genes within the embryo is likely to be determined by the transactivation of the *cis*-elements present in the 5′ flanking regions by seed expressed transcription factors. The 857 bp Bn-*FAE1.1* promoter conferred a spatially regulated expression reflecting a progressive transcriptional activation of the gene during embryo development. The promoters of the two Bn-*FAE1* genes are nearly identical in the region proximal to, and including the translation initiation codon (−475 to +20) and therefore this region may contain all the structural elements necessary for the timing and tissue specific expression of the *FAE1* genes during the maturation phase of embryo development. That the *A. thaliana FAE1* promoter shares 75 % identity with the Bn-*FAE1* promoters over the 285 bp proximal to the initiation codon rising to 83 % within the proximal 180 bp indicates the potential importance of this region.

**Fig. 7:**
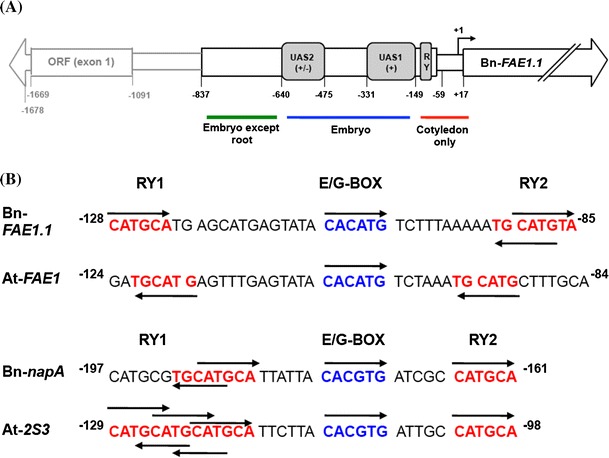
Structural organisation of the Bn-*FAE1.1* promoter. **a** Regions containing *cis*-acting elements exerting control over level and tissue specific expression as defined by deletion analyses shown in Figs. 5 and 6. UAS indicates regions exerting control over level of expression of Bn-*FAE1.1*. The *signs in parentheses* indicate the quantitative effect of each domain on level of GUS reporter expression in the embryo tissues indicated. The domains of *FAE1* expression in the embryo controlled by the corresponding promoter region are indicated *under the figure*. **b** Motifs associated with high level expression of *FAE1* and 2S napin genes in cotyledons of *B. napus* and *A. thaliana*. *Positions* are indicated relative to start of translation. *Arrows* indicate position of motif on top or bottom strands of DNA

Deletion of the region upstream of −475 caused a 27 % loss of GUS activity compared to the longest promoter sequence whereas the loss of sequences upstream of −149 caused a further 56 % loss of activity. The level of expression of Bn-*FAE1.1* in seeds is therefore determined principally by the UAS1 in the region −331 to −149 and to a lesser extent by the UAS2 in the region −640 to −475 (Fig. 7a). These two UAS regions do not contain any putative *cis*-elements in common. The 182 bp sequence of UAS1 is characterised by an elevated A/T content of 79 % within which several A/T unique islands occur, the loss of which may explain the low level of GUS activity observed in the D7 deletion. Such A/T-rich sequences have been proposed to act as general and non-specific, positive regulatory elements which enhance the activity of a proximal promoter region primarily responsible for tissue-specific expression (Sandhu et al. 1998). A/T-rich sequences have been shown to be the binding sites for HMG proteins which control protein–protein and protein–DNA interactions facilitating the formation of regulatory complexes that control transcription and recombination (Grasser 2003). The UAS2 contains putative E-boxes, located at −640 to −636 and −583 to −577, whose loss may have contributed to the lower promoter activity since these motifs have been implicated in the regulation of storage protein synthesis (Chandrasekharan et al. 2003) in developing seeds.

### The proximal promoter region of Bn-FAE1.1 controls expression in the cotyledons 

The shortest Bn-*pFAE1* fragment capable of retaining a high level of expression in the cotyledons, 73 % of that obtained with the intact promoter, encompasses the region −331 to +18. Further truncation to −149 resulted in a drastic reduction of activity to approximately 6 % of that of the intact promoter and which is restricted uniquely to the cotyledons. The promoter activity in the hypocotyl is low or absent here indicating that the region between −331 and −149 (D7 deletion) may control expression in the axis and hypocotyl. The region −331 to +18 confers a specific expression in the cotyledons and contains several candidate *cis*-elements that may constitute the binding sites for factors specifying expression in embryonic domains. Deletion or mutation of RY elements, G-box, ABRE, CCAAT box or the E-box in the promoters of genes encoding seed storage protein result in a severe loss of reporter gene activity in seeds. In the Bn-*FAE1.1* promoter two putative RY repeat motifs at −137 to −92, convergently orientated and interspaced by an overlapping E and G-box, have the potential to influence the level and expression of Bn-*FAE1* in the cotyledons and determine expression in other embryonic regions. Mutagenesis of RY elements in the β-phaseolin proximal promoter led to loss of expression in the cotyledons, whereas the proximal RY determined the expression level in the hypocotyl and radicle (Chandrasekharan et al. 2003). The Arabidopsis *FAE1* gene is a direct target of FUSCA3 recognising the RY motifs CACGTG and CATGCA (Wang and Perry 2013), confirming the importance of the RY motif in regulation of genes controlling triacylglycerol synthesis in seeds.

The E-box and G-box motifs present in the Bn-*FAE1* proximal promoters may also constitute determinants of expression in the cotyledon and in the hypocotyl since the G-box motif may constitute a functional abscisic acid responsive element. Seed VLCFA content is strongly reduced in the Arabidopsis *abi3* mutant as is the expression of *FAE1* suggesting that *FAE1* expression is controlled in part by ABA (Finkelstein and Somerville 1990; Zou et al. 1995). Neither of the Bn-*FAE1* genes possess a consensus ABRE (ACGT core) in their proximal promoters, furthermore, At-*FAE1* does not appear to be a direct target of ABI3 (Mönke et al. 2012). However, an overlapping G and E-box (ACACATG) is present in Bn-*FAE1* promoter at position −108 to −102 which resembles an ABRE-like element (Abe et al. 2003). A motif, ACACgTG, near-identical to this E/G-box is present in the proximal promoter of the carrot LEA *Dc3* gene that determines embryo specific expression and constitutes a component of an bipartite ABA responsive module (Chung et al. 2005). Mutagenesis of the G-box, identified as the functional ABA response element, together with a nearby E-box in the proximal promoter of the ß-phaseolin gene resulted in a loss of expression from the hypocotyl and severe reduction of expression in cotyledons (Chandrasekharan et al. 2003). The importance of the E and G-box motifs in the regulation of promoters of genes encoding enzymes controlling fatty acid modification has been demonstrated for *FAD2* (Kim et al. 2007) and *FAD3* (Mendes et al. 2013). Further truncation of the Bn-*pFAE1.1* to −58 to +17 resulted in an extremely weak expression evident in the cotyledons indicating that this region constitutes a minimal promoter and contains elements sufficient to promote basal transcription. The apparent absence of a consensus TATA box in proximity of TSS may be a consequence of the difficulty of prediction due the low complexity and DNA sequence degeneracy of these motifs. The CCAAT element is considered as a general eukaryotic transactivating sequence which may exhibit tissue specific regulation depending on the presence of cogent binding factors. Mutagenesis of the CCAAT box in the phaseolin gene resulted in a severe reduction of promoter activity, within specific cell types throughout the embryo (Chandrasekharan et al. 2003).

### The Bn-*FAE1* promoters possess regulatory elements in common with seed storage protein promoters

The present study suggests that regulation of Bn-*FAE1.1* expression resembles the paradigm for the regulation of expression of SSP and LEA genes. That the Bn-*FAE1.1* proximal promoter determines high level expression in the cotyledons, the main site of accumulation of storage lipid, and the more distal promoter regions serve to enhance or to extend expression is consistent with a bipartite model (Thomas 1993). The strict temporal and spatial control of gene expression within distinct embryonic domains exerted by discrete regions of the Bn-*FAE1.1* promoter (Fig. 7a) is consistent with a modular organisation of the plant embryo (Goldberg et al. 1994) where genes expressed within the embryo interact with components of gene regulatory networks unique to each embryo module or tissue, documented throughout development (Le et al. 2010; Belmonte et al. 2013). The similarities in the organisation and the presence of *cis*-elements common to the Bn-*FAE1* promoters and the promoters of genes encoding SSP imply a partially overlapping transcriptional control of the synthesis of reserves in oleo-proteinaceous seeds.

A comparison of the promoter of *FAE1* with that of the comprehensively characterised *napA* promoter of *B. napus* (Ellerstrom et al. 1996; Stålberg et al. 1996; Ezcurra et al. 1999, 2000), provides insight as to the differences in regulation of genes controlling the synthesis of seed reserve components. Sequences common to proximal promoters of the rapeseed and Arabidopsis *FAE1* genes and the *napA* storage protein genes include the E box which is present in a similar position and sequence context, interspacing two RY-elements. Alignment of the RY-G/E-RY sequences reveals overall conservation of motifs and spacing between *FAE1* and *napA* within and between species (Fig. 7b). The RY1 and 2 (CATGCA) motifs of Bn-*FAE1* are opposed on forward and reverse strands whereas these motifs read in the same reverse direction in the At-*FAE1* promoter. The RY1 and RY2 of the *napA* promoter read in the same top strand direction but the interspacing is shorter compared to the *FAE1* sequences. The RY1 and RY2 of the At-*2S3* promoter read in the same top strand direction and a short interspacing. Interestingly, the RY1 motif of the At-*2S3* promoter comprises three overlapping RY elements which may be read in forward and reverse directions. Such organisation of the RY repeat element may account in part for the stronger expression levels of *napA* compared to *FAE1* mediated via differences in binding affinity by B3 transcription factors. The motif separating the RY motifs is conserved between species but differs in sequence between *FAE1* and *napA* implying that CACATG corresponds to an E-box motif in *FAE1* distinct from the E/G-box motif (CACGTG) found in *napA*. This distinction may be important since E and G-boxes present in the proximal promoter of the *FAD2* gene of sesame have been confirmed as binding sites for a bHLH factor necessary for expression of *FAD2* (Kim et al. 2007).

A significant difference between the promoters concerns the presence of a region in the *napA* gene, the B-box, upstream of the RY-G-box, which is absent from the *FAE1* promoters. The B-box comprises a distal ABRE-like element and a proximal CA-rich motif which are both necessary for ABA-dependent seed specific expression of *napA*. The distal B interacts with the proximal B and the RY-G-box to mediate ABI3-dependent ABA *napA* activity (Ezcurra et al. 2000). This therefore raises the question as to how the Bn-*FAE1* genes respond to ABA signalling in the absence of B-box and canonical ABRE if the E/G-box in Bn-*FAE1* is not involved in ABA signal transduction. It is therefore possible that ACGT core sequences further upstream in the *FAE1* promoters are involved. Alternatively, the DRE element present in the proximal promoter may mediate ABA-dependent expression of Bn-*FAE1.1* in a manner similar to the ABA responsive *RAB17* promoter of maize (Kizis and Pagès 2002).

A further difference between the Bn-*FAE1* gene and that of the ß-phaseolin gene concerns expression in the embryo root extremity. The Bn-*FAE1.1* promoter region between −837 to −640 conferred a strong intensity of GUS staining throughout the young embryo with the exception of the radicle. This expression pattern contrasts with that of the ß-phaseolin promoter observed in transformed tobacco and Arabidopsis embryos, where expression is uniform with the intact promoter and lost from the radicle as the promoter is truncated (Chandrasekharan et al. 2003). Deletion of the UAS2 region of Bn-*FAE1.1* resulted in the extension of expression to the root extremity suggesting the loss of a negative regulatory element within this region.

### The KCS encoded by Bn-*FAE1.1* may have additional functions in planta

The finding that Bn-*FAE1.1* promoter was active in vascular tissues was unanticipated since previous reports indicated seed or embryo specific expression in Crucifers (Han et al. 2001; Rossak et al. 2001). It is unlikely that this expression resulted from the influence of upstream *B. napus* genomic sequences (chromosomal context) that were activated during integration of the promoter:reporter construct since expression was observed in all independent transformants of all deletions including the −58 to +17 minimal promoter, the latter, albeit at very low levels. Since we were able to detect the Bn-*FAE1.1* transcript in tissues other than embryo using stringent RT-PCR conditions, thus it is probable that Bn-*FAE1.1* promoter specifies this pattern of expression in contrast to that of At-*FAE1* expression restricted to the seed. Vascular expression of Bn-*FAE1.1* may therefore be constitutive and positive regulatory elements must be present within the proximal promoters. Furthermore, there are motifs present in the Bn-*FAE1.1* promoter that have been associated with expression in root in the promoters of other genes. Strong expression of the *rolD*, the rooting gene loci of the *Agrobacterium rhizogenes* root-inducing plasmid was observed in vascular tissues of plants transformed with a *rolD* promoter*::GUS* fusion in the tap root and was also present in the lateral roots and in leaf vascular tissues (Walley et al. 2008). Elements present in the *rolD* promoter occur up to six times in the Bn-*FAE1* promoters and may influence vascular expression of *FAE1*.

These results raise the question of a function for Bn-*FAE1.1* expression in vascular tissue of *B. napus*. With the caveat that the presence of the transcript does not imply the presence of the condensing enzyme there is no reason why *FAE1* expression needs to be restricted to the seed since VLCFA serve as precursors for structural and signalling lipids. We suggest that expression of Bn-*FAE1* in vascular tissue is consistent with the synthesis of elongated fatty acyl chains as precursors for suberin polymers which function as barrier lipids to impermeabilise the root and aerial vascular tissues. In accordance with this idea, we were able to show approximate coincidence of Bn-*FAE1* expression and suberin deposition in vascular tissue. We do not exclude that other KCS enzymes may also provide precursors for suberin synthesis vascular tissue in *B. napus*.

The phenomenon of impermeabilisation of aerial vasculature to our knowledge is poorly documented. The properties of suberin as an apoplastic transport barrier for water and solutes are mostly determined by the aliphatic domain comprising saturated and unsaturated aliphatic oxygenated fatty acids with chain lengths of C16–C30. Based on the continuity of expression of the Bn-*FAE1.1* gene between the aerial vascular tissues and the root vasculature detected by GUS staining together with the co-localisation of Bn-*FAE1.1* expression with the presence of suberin in roots and stems, we propose an hypothesis whereby *FAE1* genes arose via gene duplication from an ancestral *KCS* gene controlling elongated fatty acid production for suberin synthesis. In support of this idea, phylogenetic analyses in Arabidopsis, reveal that *FAE1* (KCS18) belongs to a sub-class of KCS proteins which includes KCS8 and KCS16. The genes encoding these proteins are related by ancient and recent chromosomal duplication events to genes encoding proteins belonging to a second sub-class including KCS9. KCS proteins belonging to the same or related sub-classes possess strong sequence identity and conservation of amino acids important for function (Joubès et al. 2008). KCS9 has overlapping substrate preferences with *FAE1* (KCS18) and has been implicated in the provision of C24 chain length precursors for suberin synthesis and cuticular wax as well as membrane and signalling lipids (Kim et al. 2013). *FAE1* (KCS18) also shares similar substrate preferences with KCS2 (*DAISY*, At1g04220) with respect to elongation of saturated and unsaturated fatty acids (Paul et al. 2006). DAISY provides C20:0–C24:0 acyl monomers as precursors for synthesis of cuticular wax and root suberin (Lee et al. 2009; Franke et al. 2009). It is probable that similar gene duplication and transposition events have led to neo-functionalisation of Bn-*FAE1* genes such that expression in the embryo has been acquired, whereas in contrast to Arabidopsis, expression in vascular tissues has been retained. The preferred substrates of Bn-*FAE1* are C18:0 and C18:1 (Domergue et al. 2000) which would provide similar C20:0–C24:0 acyl monomers as DAISY. If this hypothesis is valid, then expression of Bn-*FAE1.1* in vascular tissue may constitute evidence for the existence of suberin barrier lipids in aerial vascular tissues. It is clear that all vascular tissues require impermeability for function, by example, suberin is found in the bundle sheath of C4 plants separating mesophyll CO_2_ fixation from vascular bundles (Kolattukudy 2001).

That Bn-*FAE1.1* is expressed in response to wounding would be consistent with our suggestion that VLCFA produced by Bn-*FAE1* are used for suberin assembly. Suberin is deposited at wound edges as wound periderm serving to protect healthy tissue (Kolattukudy 2001) where structural reinforcement by aliphatic suberin would be expected to increase resistance to fungal attack (Lulai and Corsini 1998).

## Materials and methods

### Plant growth

*Brassica napus* seedlings (spring HEAR cultivar Lirawell), were vernalised for 4 weeks and the plants were grown on in a greenhouse under 16 h light at 22 °C and 8 h dark at 18 °C. Individual flowers on the primary inflorescence were manually pollinated and tagged on the day of pollination and the inflorescence was bagged. Siliques were collected from individual plants at different stages of seed development at intervals of 1 week after pollination. Leaf, stem, flower and siliques and seeds were collected from T3 transgenic lines for histological examination or for quantitative reporter gene assays.

### RNA isolation and RT-PCR

Total RNA was extracted from *B. napus* tissues and embryos dissected from seeds by the protocol described in Ruuska and Ohlrogge (2001). RT-PCR was performed using 100 ng of DNAase-treated RNA as template. Primers were derived from the CE7 and CE8 cDNA sequences (Barret et al. 1998) and were designed to detect gene specific transcripts (Table 1). RNA loading was controlled by PCR amplification of a fragment of the *Ribosomal Protein Large subunit 2* gene (*RPL2*) using the primers RPL25 and RPL23.

**Table 1:** Sequences of oligonucleotides used in this study

Bn-FAE1 promoter walking
FAES1: 5′ GTACTTGGACCGTCTACGATCTCC 3′
FAES2: 5′ GACGATCGCCGTTAACGGAAAGAAG 3′
S1LX3: 5′ GCAGTGCCGTCTCTTGGCCATGGC 3′
S1LX4: 5′ GCTACCTTGGCATTTGCCATGGGTCC 3′
FAED1: 5′ GCAGTGTTCCCAAGGACTATTTGTT 3′
FAED2: 5′ GTTGGTTATGACGTAATGGTAAAGG 3′
BnLH1.1: 5′ GTGGAGATAACTTCCCAACTATTTAC 3′
BnLH1.2: 5′ CTTGTAAGCTTTAGCCTTTGAGCT 3′
Gene specific RT-PCR
CE71: 5′ CTATTTTGCTCTCCAACAAGCCTG 3′
CE72: 5′ CTAGCACATCAATGACGGCTC 3′
CE81: 5′ GACGATGAGAACGGCAAAAC 3′
CE82: 5′ CTACATCGATCGGTGCTAGGC 3′
RNA quantitation control
RPL25: 5′ GTGATCGTGGTGTCCTCGCTAGAGC 3′
RPL23: 5′ GTCTGCCTTGGCAGCTGAAGCAGC 3′
Primer extension analysis
FAE17: 5′ GTGAAGATCGTCTATGGTAAGCCG 3′
FAE18: 5′ GTGGTGAAGATCGTCTATGGTAAG 3′
Promoter deletions
FAE11F3: 5′ TAACGCCTAATGGTCACCG 3′
FAE11F4: 5′ GACCTATGGACCCATGGC 3′
FAE11F5: 5′ TAGCCTATCACTGCTAAGTAC 3′
FAE11F6: 5′ AGACAGAAATCTAGACTC 3′
FAE11F7: 5′ GCACCTTTCATCGGACTACTG 3′
FAE11F8: 5′ ACGGACCACAAAAGAGGATCC 3′
FAE11R: 5′ ACGGACGTCATGACTCAGTGTGTG 3′
PCR test for nptll/GUS transgenics
Nptll
TN5FOR: 5′ CGCAGGTTCTCCGGCCGCTTGGGTGG 3′
TN5REV: 5′ AGCAGCCAGTCCCTTCCCGCTTCAG 3′
Internal control
RES FOR: 5′ GGTCAGGTTGCCTAGGAAGC 3′
RES REV: 5′ CGAGTGACACTTGATGTGAACATGC 5′
GUS
GUS2FOR: 5′ TGACGCATGTCGCGCCAAGAC 3′
GUS2REV: 5′ ATCCTTTGCCACGTAAGTCC 3′

### Isolation of Bn-*FAE1* promoters

The promoter regions corresponding to the sequence upstream of the predicted translation start site of Bn-*FAE1.1* and Bn-*FAE1.2* were isolated from rapeseed genomic DNA by a PCR walking technique (Devic et al. 1997). Two successive walks from the coding sequence into the promoter region for each gene were performed using the gene specific primers defined in Fig. 1. The resulting fragments were cloned, sequenced and contigs of 1675 and 1860 bp were assembled for Bn-*FAE1.1* and Bn-*FAE1.2* respectively.

### Bn-*FAE1* transcription start site mapping

The Transcription Start Site was mapped for each of the Bn-*FAE1* genes by Primer Extension analysis. FAE17 and FAE18 oligonucleotides (Table 1) were labelled with ^32^P via polynucleotide kinase, purified on spin columns and used to obtain the respective Bn-*FAE1.1* and Bn-*FAE1.2* specific products after reverse transcription from RNA isolated from *B. napus* immature embryos. The primer extension reaction products were resolved by electrophoresis on denaturing 5 % polyacrylamide gels alongside a sequence ladder obtained from a cloned Bn-*FAE1* promoter sequence and a ^32^P labelled pBR322 size ladder.

### Creation of promoter-deletion:reporter constructions for plant transformation

A series of fragments of sizes −854, −657, −492, −348, −166, −85 bp relative to the Adenine of the initiation codon of the coding sequence of the Bn-*FAE1.1* gene were amplified from *B. napus* genomic DNA using oligonuclotides that contained a *Pst*1 site at the 5′ extremity of the forward primer and a *Nco*1 site at the 3′ extremity of the reverse primer as DNA as defined in Table 1 with a proof-reading polymerase. The amplimers were cloned and plasmid DNA was sequenced to confirm insert identity with the Bn-*FAE1.1* promoter. The promoter fragments were digested and ligated into the pCAMBIA1381 vector containing the *uidA* (GUS) gene. The authenticity of all promoter*::uidA* fusions was verified by DNA sequencing.

### Agrobacterium-mediated transformation of *Brassica napus*

Transformation of *Agrobacterium tumefaciens* and transfection of *B*. *napus* hypocotyls (spring high erucic acid rapeseed, cultivar Lirawell) was performed as described in Nesi et al. (2009).

### Selection of transformed *B*. *napus* plants and determination of transgene copy number

A total of 33 T0 transformed lines were regenerated. Among the positive T1 plants, the rooted ones were then transferred to the greenhouse, vernalised and self-pollinated to produce T2 seeds. Twenty-four seeds were sown from each independent T2 transformant line for isolation of genomic DNA from the first leaf. Genotyping was performed on duplicate DNA samples to determine the number of T-DNA insertion loci in homozygous lines using quantitative PCR. DNA was extracted from first leaf 10 days after sowing. Duplicate PCR reactions were performed for each DNA. PCR Amplification of the *nptII* and GUS genes was performed in multiplex using the primers described in Table 1. Amplification of a single copy rapeseed gene (RES) served as positive internal control for the presence of DNA in sufficient quantity and quality. A total of 672 plants were genotyped using the protocol for transgene segregation described in Nath et al. (2007). After genotyping approximately 168 homozygous plants were obtained.

### Histochemical and quantitative analyses of reporter gene activity

Homozygous plants T3 plants were grown and 3 individual flowers were tagged per plant. Immature (25 DAP) and mature siliques were harvested and aliquots of seeds were recovered and were stored at −80 °C for activity assays. Histochemical and fluorometric assays of GUS activity were performed essentially as described by Jefferson et al. (1987). Various samples of stem sections, leaves and flowers and immature and mature seeds of the Bn-*FAE1.1* promoter:reporter transformed lines were incubated in a buffer containing 1 mM 5-bromo-4-chloro-3 indolyl-β-d-glucuronide for up to 16 h at 37 °C. Thin sections required longer incubation times of up to 16 h whereas maturing seeds were incubated for 4 h. All treated tissues were decolourised with changes of 70 % ethanol. Tissues were photographed with a binocular microscope and Zeiss Axioplan microscope and seeds were decolourised and examined in Hoyer’s solution. Results shown are representative of a total of 129 plants documented comprising up to 4 individual plants of each of 5–8 transformation events for each of the promoter deletions together with non-tranformed plants. GUS activity was quantified fluorimetrically in mature seeds. Aliquots of three seeds were homogenised in 50 mM sodium phosphate pH 7.0, 10 mM EDTA, 0.1 % Triton X-100, 0.1 % sarcosyl, 10 mM mercaptoethanol. Aliquots from the 10,000×*g*, 10 min supernatant were incubated in this buffer containing 4-methyl-umbelliferyl-β-d-glucuronide for 2 h. Initial rates of fluorescence emission at 460 nm were determined using a microplate reader. The protein content of the extracts was determined using the Bradford microassay at 595 nm to derive specific activities.

### Histology

Selected sections were stained for 30 s in 0.5 % (w/v) toluidine blue O in 0.1 M PBS, rinsed abundantly with water. Vibratome cut sections were stained for suberin in 0.1 % (w/v) berberine hemi-sulphate in distilled water for 1 h (Brundrett and Enstone 1988). The sections were rinsed in several changes of distilled water and transferred to 0.5 % (w/v) aniline blue WS in distilled water for 30 min, then rinsed as above. The sections were incubated for 5 min in a solution of 0.1 % (w/v) FeC1_3_ in 50 % (v/v) glycerine and transferred to slides for examination by fluorescence microscopy.

### Microscopy and photography

Sections were observed using a Zeiss Axiophot microscope, with UV illumination using excitation filter G 365 (365 nm peak emission), chromatic beam splitter FT 395 (395 nm) and barrier filter LP 420 (allowing wavelengths >420 nm to pass).

### DNA sequencing and analysis

Sequences were determined using DyeDeoxy Terminator cycle sequencing (Applied Biosystems) on double stranded DNA templates with an ABI 373A sequencer. Each strand was sequenced using vector and cDNA specific derived oligonucleotide primers. The cDNA sequences were identified by comparison with the NCBI database and deduced proteins were aligned with the ClustalW algorithm. The nucleotide sequence corresponding to the Bn-*pFAE1.1* and Bn-*pFAE1.2* promoter regions have been entered in the Genbank database under the accession numbers KP294338 and KP294339 respectively.

## Electronic supplementary material

Below is the link to the electronic supplementary material.

Supplementary material 1 (PPT 1687 kb)
