# Supplementary material for: Regulation of FATTY ACID ELONGATION1 expression in embryonic and vascular tissues of Brassica napus
Source: Plant Mol Biol. 2015 Mar 21;88(1):65–83. doi: 10.1007/s11103-015-0309-y (PMC4408364; doi:10.1007/s11103-015-0309-y)
Supplement: Supplementary file 1 — Supplementary material 1 (PPT 1687 kb) [file 11103_2015_309_MOESM1_ESM.ppt]

## Slide 1
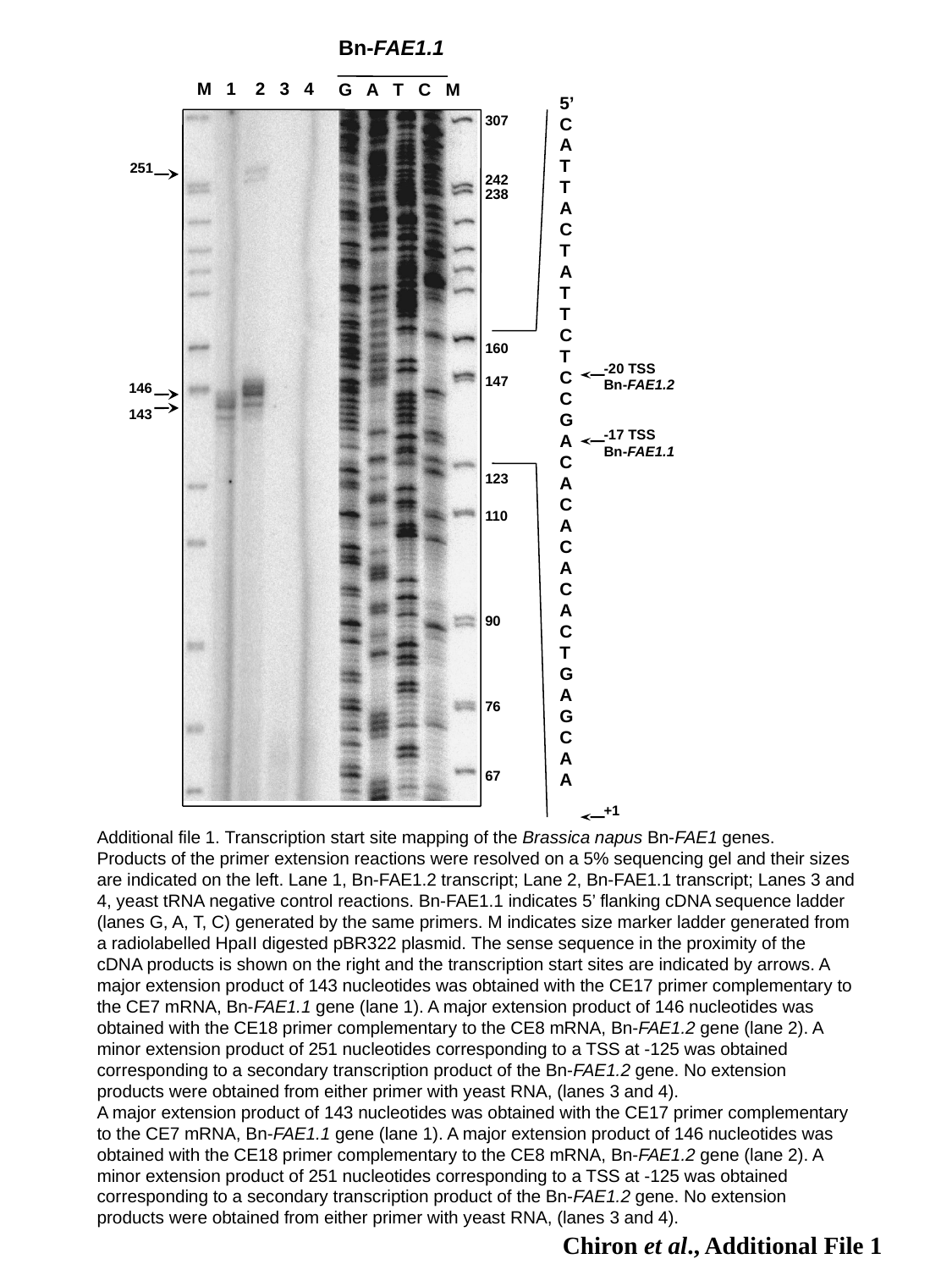

Bn-FAE1.1
 M 1 2 3 4
G A T C M
5’CATTACTATTCTCCGACACACACACTGAGCAA
307
251
242
238
160
-20 TSS Bn-FAE1.2
147
146
143
-17 TSS Bn-FAE1.1
123
110
90
76
67
+1
Additional file 1. Transcription start site mapping of the Brassica napus Bn-FAE1 genes.
Products of the primer extension reactions were resolved on a 5% sequencing gel and their sizes are indicated on the left. Lane 1, Bn-FAE1.2 transcript; Lane 2, Bn-FAE1.1 transcript; Lanes 3 and 4, yeast tRNA negative control reactions. Bn-FAE1.1 indicates 5’ flanking cDNA sequence ladder (lanes G, A, T, C) generated by the same primers. M indicates size marker ladder generated from a radiolabelled HpaII digested pBR322 plasmid. The sense sequence in the proximity of the cDNA products is shown on the right and the transcription start sites are indicated by arrows. A major extension product of 143 nucleotides was obtained with the CE17 primer complementary to the CE7 mRNA, Bn-FAE1.1 gene (lane 1). A major extension product of 146 nucleotides was obtained with the CE18 primer complementary to the CE8 mRNA, Bn-FAE1.2 gene (lane 2). A minor extension product of 251 nucleotides corresponding to a TSS at -125 was obtained corresponding to a secondary transcription product of the Bn-FAE1.2 gene. No extension products were obtained from either primer with yeast RNA, (lanes 3 and 4).
A major extension product of 143 nucleotides was obtained with the CE17 primer complementary to the CE7 mRNA, Bn-FAE1.1 gene (lane 1). A major extension product of 146 nucleotides was obtained with the CE18 primer complementary to the CE8 mRNA, Bn-FAE1.2 gene (lane 2). A minor extension product of 251 nucleotides corresponding to a TSS at -125 was obtained corresponding to a secondary transcription product of the Bn-FAE1.2 gene. No extension products were obtained from either primer with yeast RNA, (lanes 3 and 4).
Chiron et al., Additional File 1
